# Suboptimal prehospital decision- making for referral to alternative levels of care – frequency, measurement, acceptance rate and room for improvement

**DOI:** 10.1186/s12873-022-00643-3

**Published:** 2022-05-23

**Authors:** Carl Magnusson, Magnus Andersson Hagiwara, Gabriella Norberg-Boysen, Wivica Kauppi, Johan Herlitz, Christer Axelsson, Niclas Packendorff, Glenn Larsson, Kristoffer Wibring

**Affiliations:** 1 Department of Molecular and Clinical Medicine, Institute of Medicine, Sahlgrenska Academy, University of Gothenburg, SE-405 30 Gothenburg, Sweden; 2 Department of Prehospital Emergency Care , Sahlgrenska University Hospital, SE-411 04 Gothenburg, Sweden; 3 Prehospen-Centre for Prehospital Research, Faculty of Caring Science, Work Life and Social Welfare, University of Borås, SE-501 90 Borås, Sweden; 4 Department of Ambulance and Prehospital Care, Region Halland, SE-302 49 Halmstad, Sweden

**Keywords:** Patient safety, Prehospital care, Time-sensitive conditions, Decision support

## Abstract

**Background:**

The emergency medical services (EMS) have undergone dramatic changes during the past few decades. Increased utilisation, changes in care-seeking behaviour and competence among EMS clinicians have given rise to a shift in EMS strategies in many countries. From transport to the emergency department to at the scene deciding on the most appropriate level of care and mode of transport. Among the non-conveyed patients some may suffer from “time-sensitive conditions” delaying diagnosis and treatment. Thus, four questions arise:

1. How often are time-sensitive cases referred to primary care or self-care advice?
2. How can we measure and define the level of inappropriate clinical decision-making?
3. What is acceptable?
4. How to increase patient safety?

**Main text:**

To what extent time-sensitive cases are non-conveyed varies. About 5–25% of referred patients visit the emergency department within 72 hours, 5% are hospitalised, 1–3% are reported to have a time-sensitive condition and seven-day mortality rates range from 0﻿.3 to 6%.

The level of inappropriate clinical decision-making can be measured using surrogate measures such as emergency department attendances, hospitalisation and short-term mortality. These measures do not reveal time-sensitive conditions. Defining a scoring system may be one alternative, where misclassifications of time-sensitive cases are rated based on how severely they affected patient outcome.

In terms of what is acceptable there is no general agreement. Although a zero-vision approach does not seem to be realistic unless under-triage is split into different levels of severity with zero-vision in the most severe categories.

There are several ways to reduce the risk of misclassifications. Implementation of support systems for decision-making using machine learning to improve the initial assessment is one approach. Using a trigger tool to identify adverse events is another.

**Conclusion:**

A substantial number of patients are non-conveyed, including a small portion with time-sensitive conditions. This poses a threat to patient safety. No general agreement on how to define and measure the extent of such EMS referrals and no agreement of what is acceptable exists, but we conclude an overall zero-vision is not realistic. Developing specific tools supporting decision making regarding EMS referral may be one way to reduce misclassification rates.

## Background

The emergency medical services (EMS) have undergone some dramatic changes during the past few decades. Increased EMS and emergency department (ED) utilis﻿ation, changes in seeking behaviour and improved competence among EMS personnel have given rise to a shift in EMS strategies in many countries. From transporting in principle every patient to the ED by ambulance to instead assessing the patient at the scene, providing care and, depending on the assessed severity, deciding on the most appropriate level of care and mode of transport [1, 2]. There are several benefits to non-conveyance. It is considered to be an appropriate response to patients whose needs can be met at a lower level of care [3]. ED crowding has also been associated with increased short-term mortality and negative effects on the system and on the patient [4–6]. Decision-making at the scene has also been shown to reduce emergency admission rates [7]. In the EMS, non-conveyance has produced a reduction in mission time, thereby increasing availability for more urgent calls [8, 9].

In Sweden the EMS is tax financed and is organi﻿sed under one of the 21 counties with their own responsibility for the healthcare provided within the county. All of the ﻿ambulances in Sweden are comparable with advanced life support (ALS) units regarding equipment, type of assignments but also responds to lower priority assignments. All patients in contact with the emergency number (112) and where an ambulance is dispatched are assessed by at least one registered nurse (RN) according to Swedish legislation. In some of the regions a differentiated EMS is used including single responders manned with a specialist nurse and predominately allocated to non-emergent cases. To aid the RN in the patient assessment most of the EMS organisations uses a triage system, the rapid triage and treatment system (RETTS) or an equivalent system with similar structure. The RETTS includes common patient presentations and comprises of two parts, emergency signs and symptoms (ESS) and vital signs (VS). Each of those yields a colour representing patient severity from red, orange, yellow and green of which the highest colour of either the ESS or VS forms the final triage. Red and orange are considered acute processes directly whereas yellow and green can wait [10]. Assessing non-emergent patients according to the triage system ﻿i.e. the lowest triage level (green or yellow in selected cases) together with additional guidelines for inclusion/exclusion criteria to decide upon non-conveyance is used in many EMS organi﻿sations in Sweden. There is also a possibility for consultation with a physician when assessing patients at the scene, although this varies across Sweden including dispatch centre physicians, ED physicians, anaesthesiologists, cardiologists, paediatricians or primary care physicians in more rural areas. Furthermore, physician manned units within the EMS organis﻿ations have also been introduced for support at the scene and the provision of care to reduce transport to the ED. However, from an international perspective the utilisation of decision support systems in the EMS is limited regarding EMS referral [1].

A number of possibilities are available in terms of the appropriate level of care, but three main alternatives remain as the key principles: 1) Transport to hospital (directly to the ED or on different pathways directly to specialist care, for example, stroke/transient ischaemic attack (TIA), acute coronary syndrome (ACS) or hip fractures); 2) Referral to primary care or physician-manned mobile care units; 3) The patient remains at the scene with self-care advice.

The proportion of patients seen by the EMS who are candidates for options other than the ED will most likely differ from country to country and even between regions within a single country. In Sweden, actual non-conveyance rates vary substantially and have been reported to be between 10 and 20% [11–13] but figures higher than that have also been suggested for specific patient groups [14]. However, these figures do not include the emergency medical dispatch (EMD) telephone referral to alternatives other than emergency ambulance dispatch. In fact, non-conveyance rates in Sweden are lower compared with the United Kingdom (UK), where figures between 23 and 51% have been reported [15]. The variation in non-conveyance rates is explained by the EMS clinicians’ skills and competence, EMS organisational culture and support, distance to hospital and the availability of referral alternatives [3]. Previously, EMS guidelines regulated non-conveyance towards specific patient groups such as persons with hypoglycaemia. However, an increased competence level in the EMS and the amount of calls to the EMD with low-acuity ailments have led to an individual emergency nurse assessment at the scene where all patients may be eligible for other alternatives of care if assessed as not being in need of acute specialist care resources in hospital.

Conducting a patient assessment already at the scene and dividing patients into high and low risk is often a challenge and a large number of aspects have to be considered [12, 16]. Conditions such as ACS, TIA/stroke and sepsis have a number of different “behaviours” and “faces” and may be difficult to identify, particularly at an early stage. It is therefore reasonable to assume that an early assessment to either hospital care or a lower level of care will necessarily be associated with some so-called “time-sensitive conditions” being left at home or referred to primary care by mistake. This may be denoted as a “threat to patient safety”.

The term “patient safety” is defined according to the World Health Organisation as “the prevention of errors and adverse effects to patients associated with health care” [17]. Although there is a consensus that patient care should be safe, effective, and person-centred. More specific definitions aimed at a prehospital context are not discussed to the same degree. This is something that needs to be considered, but little argument about this dilemma and how to handle it can be found in the literature [18].

The aim of this paper is to discuss EMS referral to self- or primary care and the patient safety aspect of misclassifications entailed with EMS referral.

Four major questions arise:

1. How often are time-sensitive cases referred to primary care or self-care advice?
2. How can we measure and define the level of inappropriate clinical decision-making?
3. What is acceptable?
4. How to increase patient safety?

## Main text

### How often are time-sensitive cases referred to primary care or self-care advice?

The risk that patients who suffer from a time-sensitive condition will be recommended to remain at the scene or referred to primary care following a suboptimal assessment depends on a number of factors. Previous experience suggests that, in a minority of cases, the explanation may simply be that the patient did not wish to be transported to hospital [19]. Another explanation may be that the delay from the onset of symptoms to calling for the EMS was prolonged so that, although the patient was suffering from a serious disease, the time window for causal treatment, as well as the risk of serious complications, had passed [19]. A third explanation is that, conjointly with patient/relatives and the emergency physician, transport to the ED is regarded as unethical and medically unjustifiable [20].

However, in many cases, there is no obvious explanation, and the misclassification could be attributed to inadequate patient assessment due to the difficulty ruling out time-sensitive conditions at the scene. Many of these presentations refer to older patients that have atypical, vague symptoms and no deviating vital signs [11, 20–22]. This will thereby increase the risk of complications and limit the chances of a successful outcome [20, 23].

So, how often are patients with a time-sensitive condition misclassified by the EMS as appropriate for self-care advice or primary care? In a randomis﻿ed clinical trial among patients assessed by the EMS as candidates for primary care, Norberg Boysen et al. found that 2% had a final diagnosis equivalent to a time-sensitive condition [24]. However, in a much larger proportion (31%), there was in retrospect hesitation as to whether the patients were true candidates for primary care [24].

Magnusson et al. described 529 patients assessed by a single responder unit due to vague symptoms and uncertainty in the evaluation at the dispatch centre. Of them, 38% were referred to self-care advice or primary care of which 4% were hospitalised within 72 hours and required treatment in hospital [8]. Another study by Magnusson et al. reported on an urban prehospital general population where 1312 patients remained at the scene after assessment and of those 10% visited the ED within 72 hours. In total, 1% of patients who were not transported were diagnosed with a time-sensitive condition. All-cause seven-day mortality was 1%. However, after follow-up, in only five cases was the outcome deemed to have been different if the patients had been transported directly by ambulance at the initial assessment [11]. In another study from Sweden including 4853 patients with a final diagnosis of TIA/stroke who were candidates for EMS transport to hospital were 4% not directly transported to hospital after EMS assessment mainly due to vague symptoms [19]. These results correspond to those in another study in which 3% of patients with a final diagnosis of TIA/stroke were triaged by the EMS to self-care advice [25]. An unpublished retrospective study with the emphasis on patients with abdominal pain, who were assessed by the EMS and triaged to self-care advice, comprised 194 patients. Of them, 16% sought treatment for the same symptoms within 96 hours and 6% were hospitalised. Among the hospitalised patients, 3% had time-sensitive diagnoses. In another unpublished study, 2691 non-conveyed patients were included. Among them, 5% were subsequently hospitalised for the same symptoms and the seven-day mortality was < 1%. The above-mentioned Swedish studies are on a par with international literature reporting that 6.4–25.8% of non-conveyed patients accessed the ED within 72 hours and seven-day mortality rates varied from 0.3 to 6.1% [1].

### How can we measure and define the level of inappropriate clinical decision-making?

A straightforward and fairly easily accessible way to quantify inappropriate prehospital decision-making and referral is to measure what proportion of referred patients who are: 1) attending an ED within 72 hours, 2) are hospitalised within 72 hours and 3) deceased within 7 days. However, these measures are not revealing if the patient actually had a time-sensitive condition, thus they should rather be regarded as surrogate measures. Furthermore, it is obvious that the consequences of the failure to refer patients with time-sensitive conditions to the appropriate level of care will differ markedly from one another. When, for example, comparing an uncomplicated TIA with an extensive myocardial infarction, both of which can be classed as time-sensitive conditions, the risk of adverse consequences of a misclassification will differ. For this reason, only reporting the proportion of cases with a time-sensitive condition will not be sensitive enough. Some type of scoring system may be one alternative. For example, the misclassification of a time-sensitive condition, with a life-threatening complication or major sequelae or death, may receive the highest score, whereas the misclassification of a time-sensitive condition without any complication and without any sequelae may receive a lower score. A context-based dimension should also be incorporated in relation to these outcomes. It seems reasonable to differentiate death between a person in an end-of-life stage, where a senior physician has been consulted, compared with inappropriate clinical decision-making at the scene, where time to intervention is essential to the outcome.

### What is acceptable?

The Neely Conference in 2003 brought together a number of EMS experts to define a set of outcome criteria to be used when evaluating EMS triage protocols supporting alternative levels of care [26]. It was concluded that it was theoretically possible to define medical necessity based on a number of clinical criteria. However, the criteria that should be used and the rate of under-triage or misclassification that was acceptable were not agreed upon [27]. In the United States (US), the American College of Surgeons, in their optimal resources document in 2006, recommended less than 5% under-triage and a 25–35% over-triage as acceptable regarding prehospital decision-making relating to hospital transport (level-1 trauma centre or not) when assessing trauma victims at the scene [28].

In another study from the US, the EMS clinicians’ accuracy relating to patient final disposition (admission) was 79%, with a sensitivity of 72% and a specificity of 83%. The authors suggest that this is a reasonable accuracy [29], whereas others have reported an under-triage of 10% as unacceptably high, but that study dealt with whether or not the patient should remain at the scene [30]. When validating cardiopulmonary resuscitation protocols for termination at the scene, misclassification rates of less than 1% have been regarded as acceptable [31]. However, this relates to the most severe cases, i.e. patients in cardiac arrest. At the other end of the spectrum, readmission rates to the ED as an outcome measurement of the quality of care provided has been questioned. This is mainly because the cohort of patients with readmission have more chronic conditions and more multiple emergency contacts [32].

In spite of this, a vast number of these patients may receive better care with continuous care contact at primary care level in their residential homes with care provided by the responsible physician. A case-mix correction has been proposed if this measurement is used to evaluate the quality of care provided [33]. It seems reasonable to assume that a zero vision, i.e. no misclassification at all, will not be realistic. Even for patients attending the ED, a misdiagnosis and inappropriate discharge is a well-known phenomenon [34, 35], also regarding time-sensitive conditions such as myocardial infarction and sepsis [36–40]. In a meta-analysis including 15,721 patients, 9% with cerebrovascular events were missed at initial ED presentation and thus had a delayed diagnosis and were considered a non-trivial rate of misdiagnoses [41].

Although, the American college of surgeon’s agreement of rates of over-triage and under-triage concerns patients that are in fact transported to hospital and is not directly transferable to non-conveyed patients, an agreement of rates has been communicated. A similar course could be discussed for non-conveyed and conveyed patients i.e. rates of over-triage for patients transported with ambulance to the ED with no need of hospital resources and rates of under-triage for patients referred to primary care who then was rereferred to the ED with a “time-sensitive” condition. For example, a recent study from the US of missed sepsis diagnosis in the ED stated an expected rate (0.13%) and then compared with the observed rate (0.57%) for treat-and-release patients in the ED [42].

What is an acceptable misclassification rate is also likely to differ between diagnoses and between different countries and medical systems due to differences in medical legislation.

Transporting all patients to ED naturally would entail a zero misclassification rate regarding EMS referrals to non-hospital care. However, such an approach would result in an increase of unnecessary EMS transports to the ED, an increased ED attendance resulting in ED crowding and thus a threat to patient safety [4]. Furthermore, allocated ambulances with non-emergent patients with conditions that could be managed in primary care could also be considered a suboptimal use of EMS resources. Thus, based on comments above the question what is acceptable is at present best addressed with the answer “as low as possible”.

### How to increase patient safety?

There are a number of ways to improve the quality of prehospital triage and reduce the risk of misclassifications. One alternative is to develop decision support tools with support from machine learning (ML) techniques in order to improve the quality of the initial assessment. One approach is prediction of a certain diagnosis. In a systematic review including 16 million patients with different diagnosis such as sepsis, cardiac arrest, myocardial infarction, acute ischaemic heart disease, major adverse cardiac event, infection, traumatic brain injury as well as outcomes on mortality, hospital admission, therapeutic intervention have shown promising results in both diagnostic and prognostic prediction. The ML models outperformed most of the usual care and the ML models showed a better discrimination compared to any usual care in the ED ie. triage systems, scoring systems or clinical judgment [43]. Studies on predicting outcomes such as hospital admission, intensive care unit admission and mortality of which the ML model outperformed usual decision support tools ie. dispatch priority at the dispatch centre, National Early Warning Score (NEWS), Modified Early Warning Score (MEWS) and different triage systems such as the Emergency Severity Index﻿ [44, 45]. Such models should be implemented in the EMS supporting the clinician in the decision-making process and thus give recommendation on level of care, when to consult a specialist physician and/or direct transport to hospital. It is also desirable that these tools learn from mistakes in order to improve. One challenge with the deployment of ML models is for example how to act with an iterative process of the model and retraining models with new data. Furthermore, publicly open source data of these models is also preferrable to avoid bias and could also increase knowledge of the model, which data that is included and also model limitations [46].

It is probably also important to advertise the severe mistakes so that all the colleagues in the team learn from them. A morbidity and mortality conference which aims to revisit errors in order to give an opportunity for everyone to learn and reflect is common in hospitals [47].

It might be possible to create a register of severe mistakes that are made in the prehospital setting. A register of this kind should be introduced at national level, to increase the visibility of the aggregate of misclassifications rather than local events. One of the most common ways to gather data in the healthcare system regarding threats to patient safety is to use incident-reporting systems [48]. This method requires the staff to recognise or pay attention to a mistake or a near mistake that has been made which could lead to misleading frequencies due to underreporting [49]. Another method for gathering data relating to severe mistakes is the use of a trigger tool. A trigger tool could detect adverse events up to ten times more often than a traditional reporting system. This was shown in a study conducted in American hospitals [50]. To our knowledge, a trigger tool adapted for road-based EMS is not available, but a patient safety project is under way with the development of a national trigger tool tailored to suit the Swedish EMS. Furthermore, it is necessary to realise that a single method to measure patient safety will not be sufficient, which is highlighted by Vincent et al. [51]. The events that threaten patient safety have multiple dimensions, including, for example, the work environment or different patient barriers [52], thereby highlighting the need for different methods when conducting research on the topic. The use of trigger tools could therefore be accompanied by different approaches, such as the use of qualitative designs with interviews of patients and staff regarding safety issues from their point of view and context. By itself, the data gathered by different methods will not improve patient safety, but it will serve as a foundation for organisational improvements and educational efforts for the staff.

Finally, effective collaboration with primary health care [53], as well as hospital facilities, is of the utmost importance. Important aspects regarding the latter are education, clinical support, feedback and the effective use of digital support. Attempts are being made to transfer advanced medical information (e.g. blood samples) from the EMS to a higher level of medical skills and competence via a link for consultation.

## Conclusions

The EMS has changed from being mainly a transport organisation to a larger extent focus on patient assessment and referral to different level of care depending on the assessed severity. We conclude that this shift has given rise to substantial number of patients being referred to non-hospital care, including a small proportion of patients with time-sensitive conditions. This poses a threat to patient safety. However, it a difficult task to define and measure the extent of such inappropriate EMS referral given the complexity of the phenomenon. There is no general agreement of what is an acceptable misclassification rate and this need to be handled by the scientific and clinical EMS community. We conclude a zero vision is not realistic and at present one simply may say “as low as possible”. Developing specific tools supporting decision making regarding EMS referral may be one way to reduce misclassification rates and thereby increase patient safety.

## Data Availability

Not applicable.

## Abbreviations

ACS: Acute coronary syndrome
ALS: Advanced life support
ED: Emergency department
EMD: Emergency medical dispatch
EMS: Emergency medical service
ESS: Emergency signs and symptoms
MEWS: Modified early warning score
ML: Machine learning
NEWS: National early warning score
RN: Registered nurse
RETTS: Rapid emergency triage and treatment system
TIA: Transient ischemic attack
UK: United Kingdom
VS: Vital signs
US: United States
